# Molecular alterations in basal cell carcinoma subtypes

**DOI:** 10.1038/s41598-021-92592-3

**Published:** 2021-06-24

**Authors:** Lucia Di Nardo, Cristina Pellegrini, Alessandro Di Stefani, Francesco Ricci, Barbara Fossati, Laura Del Regno, Carmine Carbone, Geny Piro, Vincenzo Corbo, Pietro Delfino, Simona De Summa, Maria Giovanna Maturo, Tea Rocco, Giampaolo Tortora, Maria Concetta Fargnoli, Ketty Peris

**Affiliations:** 1 Dermatologia, Dipartimento di Medicina e Chirurgia Traslazionale, Università Cattolica del Sacro Cuore, Roma, Italy; 2 Dermatologia, Dipartimento di Scienze Mediche e Chirurgiche, Fondazione Policlinico Universitario A. Gemelli IRCCS, Roma, Italy; 3 Dermatology, Department of Biotechnological and Applied Clinical Sciences, University of L’Aquila, L’Aquila, Italy; 4 Dermatology, Istituto Dermopatico Dell’Immacolata-IRCCS, Roma, Italy; 5 Oncologia Medica, Dipartimento di Scienze Mediche e Chirurgiche, Fondazione Policlinico Universitario A. Gemelli IRCCS, Roma, Italy; 6 Oncologia Medica, Dipartimento di Medicina e Chirurgia Traslazionale, Università Cattolica del Sacro Cuore, Roma, Italy; 7 Department of Diagnostic and Public Health, University of Verona, Verona, Italy; 8 Molecular Diagnostics and Pharmacogenetics Unit, IRCCS-Istituto Tumori “Giovanni Paolo II”, Bari, Italy

**Keywords:** Cancer, Genetics, Molecular biology, Pathogenesis

## Abstract

A number of genes have been implicated in the pathogenesis of BCC in addition to the Hedgehog pathway, which is known to drive the initiation of this tumour. We performed in-depth analysis of 13 BCC-related genes (*CSMD1, CSMD2, DPH3* promote*r, PTCH1, SMO, GLI1, NOTCH1, NOTCH2, TP53, ITIH2, DPP10, STEAP4, TERT* promoter) in 57 BCC lesions (26 superficial and 31 nodular) from 55 patients and their corresponding blood samples. *PTCH1* and *TP53* mutations were found in 71.9% and 45.6% of BCCs, respectively. A high mutation rate was also detected in *CSMD1* (63.2%), *NOTCH1* (43.8%) and *DPP10* (35.1%), and frequent non-coding mutations were identified in *TERT* (57.9%) and *DPH3* promoter (49.1%). *CSMD1* mutations significantly co-occurred with *TP53* changes (*p* = 0.002). A significant association was observed between the superficial type of BCC and *PTCH1* (*p* = 0.018) and *NOTCH1* (*p* = 0.020) mutations. In addition, *PTCH1* mutations were significantly associated with intermittent sun exposure (*p* = 0.046) and the occurrence of single lesions (*p* = 0.021), while *NOTCH1* mutations were more frequent in BCCs located on the trunk compared to the head/neck and extremities (*p* = 0.001). In conclusion, we provide further insights into the molecular alterations underlying the tumorigenic mechanism of superficial and nodular BCCs with a view towards novel rationale-based therapeutic strategies.

## Introduction

Basal cell carcinoma (BCC) is the most common epithelial skin cancer in Caucasian population^1, 2^, with 2.75 million cases per year estimated by the World Health Organization^3^. Incidence of BCC is rising of 5% per year in Europe and 2% annually in USA^4, 5^. BCCs are a heterogeneous group of tumours ranging from thin superficial papules or plaques and nodules, sometimes ulcerated, to the sclerosing/morpheic, basosquamous and infiltrating types^4, 6–8^. The superficial and nodular BCCs are non-aggressive, slow-growing lesions with low risk of recurrence, that account for 80% of BCC lesions, thus representing a growing healthcare issue^9, 10^.

The aberrant activation of the Hedgehog (Hh) pathway plays a pivotal role in the pathogenesis of BCC. In physiological condition, the Hh signalling is involved in cell type differentiation and proliferation, adult tissue homeostasis and repair with a fundamental mitogenic and morphogenic role^9^. In the absence of input signals, the twelve-pass transmembrane protein Patched 1 (PTCH1) lies in the cilia where it inhibits the Hh pathway activator G-protein coupled receptor Smoothened (SMO), thus blocking pathway activity. The binding of Hh ligands to PTCH1 results in activation of SMO allowing the release of the glioma-associated transcription factors (GLI) from their negative regulator Suppressor of fused^11^. The subsequent translocation of activated GLIs into the nucleus initiates the transcription of GLI-targeted genes involved in cell proliferation, cell cycle regulation, apoptosis, angiogenesis and self-renewal^12^. *PTCH1* inactivating mutations have been identified in 70–90% of BCCs, while 10–20% of BCC lesions harbour activating mutations in the *SMO* gene^13^. Alterations in the cell cycle regulator *TP53* gene are the second most prevalent tumorigenic event found in 44–65% of BCCs^14, 15^. Exome sequencing-based studies have shown that sporadic BCCs exhibit one of the highest prevalence rates of somatic mutations of all cancers (65 mutations/megabase), involving a number of additional Hh-unrelated genes^14–17^. These investigational studies revealed new potential BCC driver genes, although their contribution to the genetic network underlying tumorigenesis and tumour evolution is not yet completely explained.

We examined the molecular alterations across 13 genes, selected on the basis of their potential role in the pathogenesis of superficial and nodular tumour subtypes, in order to provide further insights into the molecular sub-classification of BCC lesions.

## Results

### Study cohort

Fifty-seven sporadic BCCs of 55 patients (27 males and 28 females; median age at diagnosis: 70 years, range 32–93 years) were included in the study. Demographic characteristics and clinico-pathologic features of patients and BCC lesions are illustrated in Table 1. Thirty-one patients had one BCC, 11 patients two BCCs and 13 patients > 2 BCCs. Anatomical sites of BCC lesions included the head/neck region (15/57, 26.3%), trunk (38/57, 66.7%) and extremities (4/57, 7.0%). Histopathologic examination showed that 26/57 (45.6%) were superficial BCCs and 31/57 (54.4%) nodular, with 47/57 BCCs (82.5%) having no ulceration.

**Table 1:** Demographic and Clinical-pathological features of patients and tumours.

Characteristics of patients		Patients N = 55 (%)
Age at diagnosis (years)	Median (range)	70 (32–93)
	< 70	27 (49.1)
	≥ 70	28 (50.9)
Sex	Male	27 (49.1)
	Female	28 (50.9)
Skin type	I/II	27 (49.1)
	III/IV	28 (50.9)
Solar Lentigos	Absent	7 (12.7)
	Mild	23 (41.8)
	Moderate/severe	25 (45.5)
History of sunburn	No	19 (34.5)
	Yes	36 (65.5)
History of other cutaneous neoplasia	No	46 (83.6)
	Yes	9 (16.3)
Professional UV exposure	No	33 (60.0)
	Yes	22 (40.0)
Immunosuppression	No	50 (91.0)
	Yes	5 (9.0)

BCC feature		Tumours N = 57 (%)
BCC number	Single	31 (56.4)
	Multiple	24 (43.6)
Anatomical site	Head and neck	15 (26.3)
	Trunk	38 (66.7)
	Extremities	4 (7.0)
Histopathological subtypes	Superficial	26 (45.6)
	Nodular	31 (54.4)

### Mutational screening

**Figure 1:**
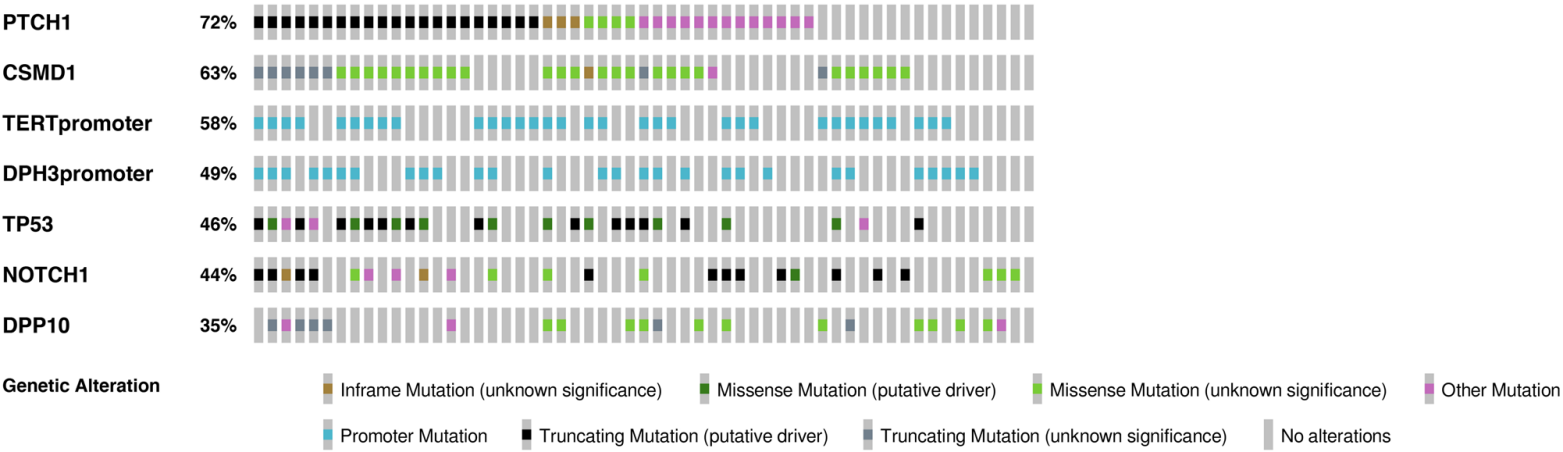
Distribution of somatic variants across the mutated genes. Each row represents a gene and each column corresponds to individual samples. When multiple mutations were present in a gene, only the pathogenetic or probable pathogenetic mutation was considered and shown in the diagram generated with Oncoprinter in cBioPortal tool.

We analysed the mutational profile of 13 cancer genes (*CSMD1, CSMD2, PTCH1, SMO, GLI1, NOTCH1, NOTCH2, TP53, ITIH2, DPP10, STEAP4, DPH3* promoter and *TERT* promoter) (Supplementary Table S1) in a total of 57 BCCs, including superficial and nodular subtypes. Somatic variants were detected across 7 genes (*PTCH1, CSMD1, TP53, NOTCH1, TERT* promoter, *DPH3* promoter, *DPP10*), with the 98.2% (56/57) of BCCs showing at least one alteration (Fig. 1). Among all coding somatic mutations, 56% (135/241) were not previously published or reported in online variant databases. In silico prediction of protein functional effect and classification of novel variants are detailed in Supplementary Table S2. As for the 44% (106/241) of already reported variants, 69/106 (65.1%) were classified as pathogenic/likely pathogenic/oncogenic/likely oncogenic, 22/106 (20.8%) were variant of uncertain significance (VUS), and 15/106 (14.1%) were benign/likely benign/polymorphism.

Overall, 81.0% of single-nucleotide variants (SNVs) were C > T changes, consistent with UV-induced mutagenesis.

**Figure 2:**
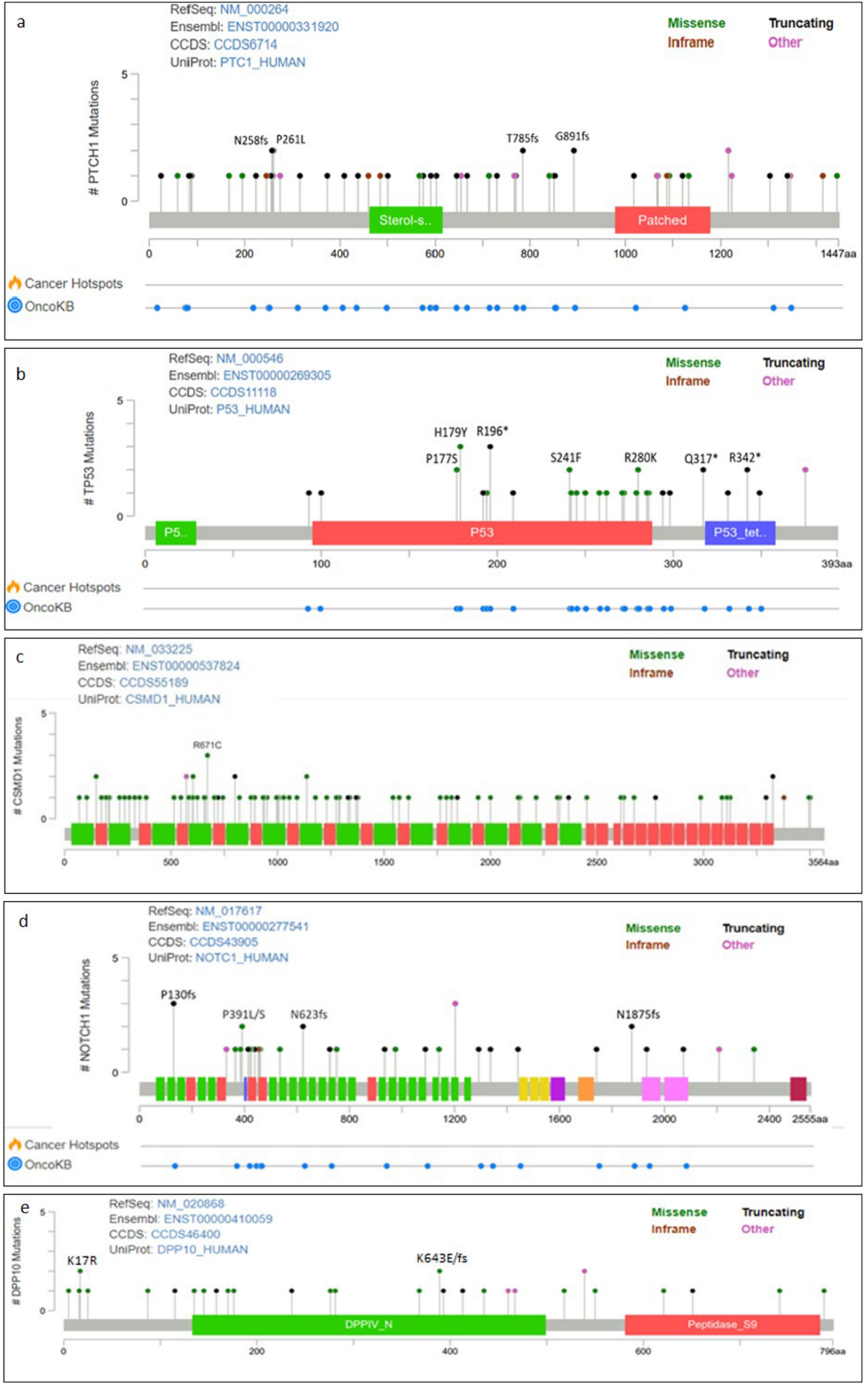
Mutational profiles interpreted with protein annotation. The protein figure was generated with MutationMapper in cBioPortal tool. Alterations occurring two and/or more times are specified in the protein domains. (**a**) *PTCH1*: the sterol-sensitive and Patched family functional protein domains are represented by green and red boxes, respectively. (**b**) *TP53*: the transactivation, DNA-binding and tetramerization domains are represented by green, red and blue boxes, respectively. (**c**) *CSMD1*: the CUB and SUSHI domains are represented by green and red boxes, respectively. (**d**) *NOTCH1*: the EGF-like domains are represented by green boxes. (**e**) *DPP10*: the dipeptidyl peptidase N-terminal domain is represented by green box.

*PTCH1* mutations were found in 41/57 (71.9%) BCCs, with 16/57 (28.1%) lesions carrying more than one *PTCH1* variants. Overall, 63 variants in the *PTCH1* gene were identified, distributing within the entire coding sequence with no specific hotspot region (Fig. 2a). The most frequent nucleotide change was the C > T transition identified in 18/57(31.6%) tumours, followed by the CC > TT tandem variants in 8/57 (14.0%) and C > A mutations in 3/57 (5.3%) tumours. The majority (27/63, 42.9%) were truncating mutations, including 10 frameshift deletions and/or insertions and 17 nonsense mutations. All the identified truncating variants were included as likely oncogenic for clinical significance and likely loss-of-function for biological effect in the OncoKB database^21, 22^. In addition, missense mutations accounted for 20.6% (13/63) of *PTCH1* variants. Among the identified *PTCH1* missense mutations, the p.R195K and p.P568L are known for their pathogenic significance. Finally, 9 splice sites mutations (8/63, 14.3%) were found, 3 of which were not previously published or reported in online variant databases and predicted to be pathogenic based on the dbNSFP v4.1 applied prediction tools (Supplementary Table S2).

*TP53* mutations were found in 26/57 (45.6%) BCCs, with hot spot positions including p.H179, p.S241, p.G245 and p.R280 (Supplementary Table S2)^24^. Single nucleotide missense variants clustering in the DNA binding domain (95–288 ammino-acid residues) were the most prevalent (15/30, 50.0%), and truncating mutations (all nonsense except for one frameshift deletion) represented 33.3% (10/30) of alterations (Fig. 2b).

In addition, mutations in *CSMD1* gene were identified in 36 of 57 (63.2%) BCCs, with missense mutations being the most prevalent (68/85, 80.0%). More than one *CSMD1* alterations was found in 22/57 (38.6%) tumours, and 5/57 BCCs (8.8%) harboured more than six *CSMD1* somatic mutations. All the identified *CSMD1* point mutations were unknown for oncogenic significance. However, the dbNSFP v4.1 tool predicted that 32.9% (28/85) of *CSMD1* alterations were deleterious for protein function (Supplementary Table S2), including the most recurrent p.R671C (c.2011C > T) amino-acid change, which was detected in 3/57 (5.3%) BCC lesions (Fig. 2c).

*NOTCH1* mutations were found in 25/57 (43.8%) BCCs. In total, we detected 35 *NOTCH1* mutations, including truncating (14/35, 40.0%) and missense variants (13/35, 37.1%). Of the 35 identified mutations, 5 (5/35, 14.3%) referred to a COSMIC identifier (Supplementary Table S2) and 16 (16/35, 45.7%) are known oncogenic or predicted oncogenic according to the OncoKB database (Fig. 2d).

Mutations in *DPP10* were detected in 35.1% (20/57) of BCCs, with missense variants being the most frequent mutational event (20/30, 66.7%) (Fig. 2e).

Eight *TERT* promoter mutations were found in 33/57 (57.9%) BCC lesions. The most frequent somatic change was the -146 C > T, which was detected in 19/57 BCCs (33.3%), followed by the -124 C > T transition and the -138/139 CC > TT tandem variation accounting for 8.8% (5/57) and 5.3% (3/57) of BCC lesions, respectively. Supplementary Table S3 illustrates all *TERT* mutations.

Overall, 9 *DPH3* promoter mutations were identified in 49.1% (28/57) of BCCs. The most prevalent variant was the -121C > T transition (19/57, 33.3%), followed by the -122 C > T (4/57, 7.0%), -125 C > T (2/57, 3.5%) and -150 C > T (2/57, 3.5%) (Supplementary Table S3).

### Statistical analysis of the mutational profile

We performed pairwise exclusivity and co-occurrence analysis for the 7 identified mutated loci (*DPP10, CSMD1, PTCH1, NOTCH1, TP53, TERT* promoter, *DPH3* promoter) and found significant concurrent variants of *TP53* gene with *CSMD1* (*p* = 0.002), *PTCH1* (*p* = 0.011) and *DPH3* promoter (*p* = 0.006) (Fig. 1). However, after Benjamini–Hochberg false discovery rate (FDR) correction, only the association of *CSMD1* variants with the *TP53* mutation rate remained significant (*q* = 0.045).

By evaluating the distribution of somatic alterations within 7 different mutated genes across 57 BCC lesions, we identified 43 different combinations. Among them, the two most frequent (4/57; 7.0%) were: 1) concurrent *PTCH1* and *TERT* promoter mutations in a setting of wild type *DPP10, CSMD1, NOTCH1, TP53* and *DPH3* promoter genes, BCCs, and 2) the coexistence of *DPP10, CSMD1, PTCH1, NOTCH1, TP53, TERT* promoter and *DPH3* promoter mutations. None of these two most frequent combinations were significantly associated with superficial or nodular subtypes (*p* = 0.117).

Focusing on the analysis of single mutated gene according to the specific BCC subtypes, *PTCH1* and *NOTCH1* mutations were found significantly associated with superficial BCCs (*p* = 0.018 and *p* = 0.020, respectively). In details, *PTCH1* variants were 1.6 times (OR = 5.537, 95% CI = 1.367–22.43) and *NOTCH1* mutations 2.0 times more frequent (OR = 4.457, 95% CI = 1.304–15.24) in superficial than in nodular BCCs.

**Figure 3:**
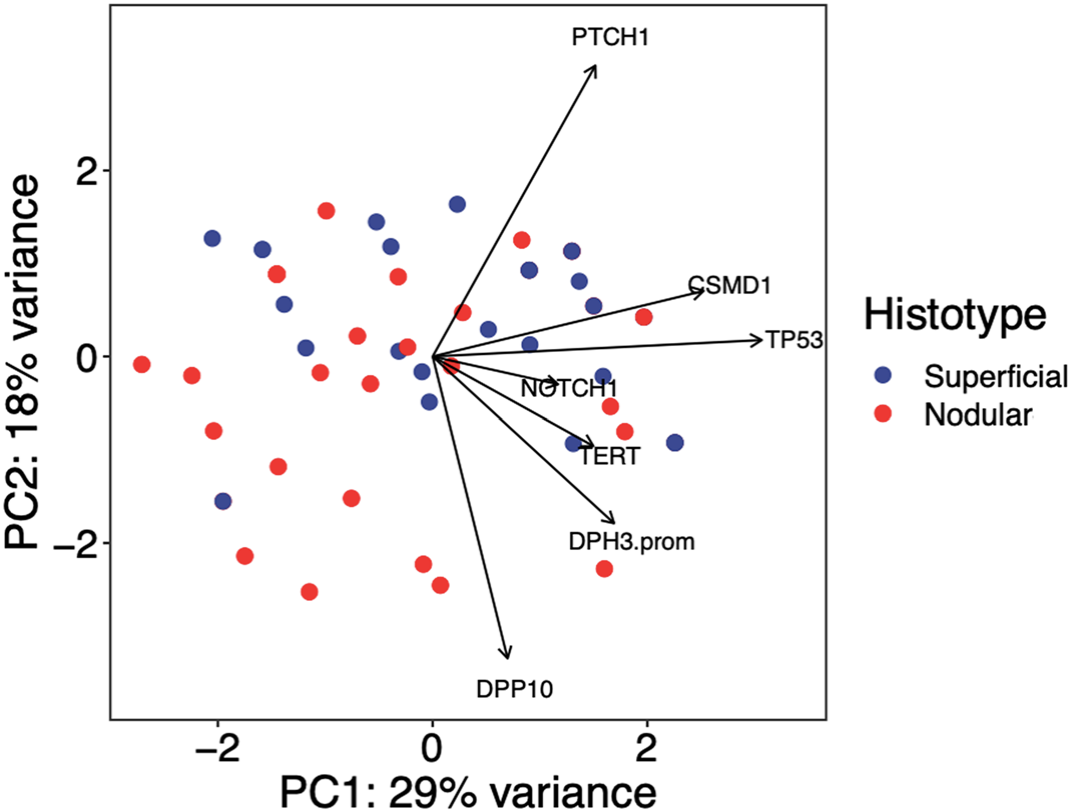
Two-dimensional principal component analysis (PCA) of genetic variations among superficial and nodular subtypes. Each sample was characterized as a distinct dot in a vector space. *PTCH1* mutations characterized the superficial BCC types that clustered on the top right of the graph (PTCH1 arrow). Over 47% of the variance in genetic profile data sets were accounted for by the first two components: PC1 (29%) and PC2 (18%).

The Principal Component Analysis (PCA) multivariate approach confirmed a significant association between *PTCH1* mutations and the superficial BCC subtypes that were recognized as genetically similar group for *PTCH1* mutations in a separate cluster of the PCA diagram (Fig. 3).

The analysis of the mutational status according to patients and tumour characteristics revealed that *PTCH1* mutations were significantly associated with intermittent sun exposure (*p* = 0.046), and with the occurrence of single BCC lesions (*p* = 0.021), and *NOTCH1* mutations were more frequent in BCCs arising on the trunk compared to the head/neck and extremities (*p* = 0.001).

## Discussion

Our in-depth analysis was focused on a panel of 13 genes potentially associated to BCC tumorigenesis. We showed a high prevalence of Hh pathway mutations and a high rate of mutations in *CSMD1*, *NOTCH1* and *DPP10* genes, and in *TERT* and *DPH3* promoter regions. Interestingly, *NOTCH1* and *PTCH1* mutations were significantly more frequent in superficial than in nodular BCCs, and *CSMD1* mutations occurred along with *TP53* changes. *PTCH1* alterations were significantly associated with intermittent sun exposure and with the development of a single BCC, while *NOTCH1* mutations with location on the trunk.

In line with previous studies^14–17, 25, 26^, 98.2% of BCCs showed at least one alteration among the analysed genes. *PTCH1* and *TP53* mutations were found in 71.9% and 45.6% of BCCs, respectively, being UV-fingerprint mutations (C > T and CC > TT transitions) the most common. Moreover, multiple *PTCH1* and *TP53* mutations were found in 28.0% and 17.6% of BCC lesions, respectively. We identified eight *PTCH1* splice site mutations including the c.1347 + 1G > A and c.1216-2A > T, which have been recently recognized as novel *PTCH1* variants^27^, probably pathogenic according to Associations for Clinical Genetic Science and American College of Medical Genetics and Genomics criteria^28, 29^. Notably, a significant association was found between *PTCH1* mutations and the superficial BCC type that was further confirmed by the PCA multivariate statistical approach. This is in line with evidences that topical imiquimod, which has been shown to negatively regulate Hh signalling^30^, is a successful treatment for superficial BCC. Moreover, a clinical trial is investigating the efficacy of topical patidegib, a novel topical Hh inhibitor, to decrease the number of surgically eligible BCC lesions in patients with multiple BCCs (NCT04155190)^31^. We found no mutations in *SMO* gene in our BCC samples, in contrast with the 10–20% frequency rate previously described^13–17^. Notably, this discrepancy might reflect clinical differences in the patient population: in our study only treatment-naïve patients have been included in the analysis while previous study showed a 2.2-fold higher frequency of *SMO* mutations in vismodegib-resistant compared to treatment-naïve sporadic BCCs (P = 5 × 10 − 2, Fisher’s exact test)^14^.

In addition to the established BCC-associated genes, we identified high frequency of mutations in *CSMD1*, *NOTCH1*, *DPP10*, *TERT* promoter and *DPH3* promoter genes. *CSMD1* gene, encoding an inhibitor of the complement system, is believed to act as a tumour suppressor gene whose functions seems to be inactivated in many cancers^32–35^. Consistently with previous data^14, 15^, we detected *CSMD1* somatic mutations in 63.2% of BCCs, thus representing the second most prevalent mutated genes after *PTCH1.* In addition, 38.6% of BCCs harboured more than one *CSMD1* alterations. It is conceivable that, under the selective pressure during cancer development, multiple *CSMD1* variants reside in separate subclones within the same tumour population. Notably, we found a significant association of *CSMD1* alterations with *TP53* mutation rate, which was previously described in mucosal head and neck squamous cell carcinoma^33^. The increased cellular proliferation driven by *TP53* loss, might provide *CSMD1* mutant clone to sufficiently expand.

NOTCH signalling pathway in physiological condition plays a critical role in the regulation of cell differentiation, self-renewal and homeostasis primarily controlling the interplay between adjacent cells^36^, while in cancer can function as either an oncogene or tumour suppressor gene depending on the cell type and context^37, 38^. Shi et al.^39^ demonstrated that NOTCH pathway activity is suppressed in BCCs highlighting its tumour suppressor function in human epithelial malignancies. Moreover, *NOTCH1* loss-of-function mutations were found to specifically promote tumour persistence in sporadic BCCs suggesting therapeutic restoring of the *NOTCH* tumour-suppressor function as a potential approach to eradicate persistent tumour cells^40^. Previous reports found *NOTCH1* loss-of-function alterations (missense, truncating or loss of heterozygosity) in 30–50% of BCCs^14, 15, 25^. In our study, *NOTCH1* mutations were found in 43.8% of BCC lesions and were mainly inactivating alterations, which support the tumour-suppressor role of *NOTCH1* in BCC tumorigenesis. Interestingly, we found that *NOTCH1* mutations were 2-times more frequent in superficial BCCs than in nodular subtypes, suggesting that tumorigenic pathways may differ across BCC subtypes.

The *DPP10* gene encodes a trans-membrane protein, which regulates potassium channels activity involved in cell proliferation and apoptosis^41^. We found mutations of *DPP10* gene in 35.1% of BCCs, while a previous study on 12 BCCs showed mutations in 75% of cases^15^. Such difference in frequency rate might be due to the sample size or different subtypes of BCC lesions analysed.

Noncoding somatic mutations of *TERT* promoter are emerging to have a crucial pathogenetic role in BCCs. *TERT* non-coding mutations were previously identified in 39–74% of BCCs and considered to contribute to telomeres length maintenance in cancer^26, 42–48^. In addition, *TERT* promoter mutations in BCCs were associated with shorter telomere and increased transcription of the telomerase reverse transcriptase subunit^13, 26^. It is thought that the genome instability caused by critically short telomeres promotes telomerase upregulation, thus sustaining cell proliferation and tumorigenesis^49^. In the present study, *TERT* promoter mutations were found in 57.9% of tumours although they were mutually exclusive.

We also identified *DPH3* promoter mutations in 49.1% of BCCs, being the -121 C > T and -122 C > T transitions the most frequent ones. Mutations in the *DPH3* promoter were previously described in 42% of BCCs.^42^ In a recent study, 73/191 (38.2%) BCCs were found to carry *DPH3* promoter mutations that were significantly more frequent in BCC patients with a clinical history of cutaneous neoplasms^24^. However, the effect of *DPH3* promoter mutations on transcription of adjacent or distant genes remains currently unclear^42^. The exclusive identification of mutations at dipirimidinic sites into *DPH3* and *TERT* promoter further highlights the role of UV-induced DNA damage in BCC tumorigenesis.

Additional in vivo studies evaluating the function of the novel variants (56%), of which 22.2% with uncertain significance, are needed to clarify their implications in BCC tumours.

In conclusions, our study provides further insights into the molecular alterations underlying the tumorigenic mechanism of superficial and nodular BCCs showing that additional genes and pathways beyond *PTCH1*-axis might contribute to BCC development and progression.

## Material and methods

### Patients and tumour samples

Sporadic BCC tumour tissues and matched blood samples were collected at the Institute of Dermatology, Catholic University—Fondazione Policlinico Universitario A. Gemelli-IRCCS, (Rome, Italy) and at the Dermatologic Clinic of the University of L’Aquila (L’Aquila, Italy) from January 2015 to July 2017 with complete medical records. Examples of superficial and nodular BCC lesion included in our study are reported in Supplementary Fig. S1. Informed consent was obtained from all patients after study approval by the local ethical committee (IRB number:15272/14, Ethical Committee Fondazione Policlinico Universitario A. Gemelli-IRCCS) and the research was performed in accordance with relevant guidelines and regulations stated in the Declaration of Helsinki. Patients and tumours characteristics are illustrated in Table 1. Only superficial and nodular types of BCCs, histologically reviewed by a single histopathologist, were included in the study.

During surgical excision, a 4-mm intra-tumoral punch biopsy specimen was obtained and stored in RNA later solution at -20 °C. DNA was extracted from fresh-frozen tumour samples using Qiagen All Prep-DNA/RNA/miRNA extraction kit (Qiagen, Hilden, Germany) after tissues homogenization in a Precellys24 homogeniser (Bertin instruments, Montigny-le-Bretonneux, France). DNA from whole blood was purified using Qiagen QIAamp Blood Midi Kit (Hilden, Germany) and quantified by Qubit dsDNA HS Assay Kit (ThermoFisher Scientific, Waltham, MA USA) on Qubit 2.0 Fluorometer instrument (Invitrogen, Carlsbad, CA, USA). Genomic DNA at least 10 ng/μL concentrated was subjected to library preparation.

### Panel design and library preparation for NGS sequencing

Overall, 12 genes (*CSMD1, CSMD2, DPH3* promoter*, PTCH1, SMO, GLI1, NOTCH1, NOTCH2, TP53, ITIH2, DPP10, STEAP4*) were selected on the basis of a comprehensive literature search and interrogation of different bioinformatics sources like the Catalogue of Somatic Mutations in Cancer (COSMIC) and The Cancer Genome Atlas (TCGA), taking into account the differences in the mutational profile between specific BCC subtypes.The custom gene-panel was designed by using Ion AmpliSeq Designer tool.

Library preparation was performed using the Ion Ampliseq Library kits 2.0 with 20 ng of DNA as input. After clonal amplification on the Ion One Touch2 System and subsequent enrichment on the Ion One Touch ES, libraries were sequenced on the Ion Torrent Ion PGM System (Thermo Fisher Scientific, Waltham, MA USA).

### Variant calling analysis

Tumour-germline paired sequencing data were primarily analysed by using IonTorrent Suite Software for base calling and alignment to the hg19 human reference genome from UCSC Genome Browser^18^. Raw data were then processed for somatic variant calling and annotation by following the VarDict workflow in paired samples (tumour/normal) analysis mode. The allele frequency and mapping quality thresholds were set at 0.05 and 30, respectively. Only the variants recognized as "StrongSomatic" were selected. To reduce the false positive rate during somatic mutation calling, several steps were used in order to retain only the high-quality variants with a minimum depth of total coverage ≥ 300 reads and each variant coverage ≥ 20 reads. Filtered variants were functionally annotated using Annovar software version 2018 Apr 16^19^.

### Prediction tools analysis

All the identified cancer genomics variants were evaluated by using the cBioPortal for Cancer Genomics tool^20^, which includes the OncoKB precision oncology knowledge database^21, 22^ for investigation of clinical significance, biological effects and treatment implications of specific cancer gene alterations. For in silico prediction of protein functional effect of variants we referred to the dbNSFP v.4.1 database (database of Human Non-synonymous SNVs and their Functional Predictions), which includes SIFT, PolyPhen2 HDIV, PolyPhen2 HVAR, LRT, MutationTaster, MutationAssessor, FATHMM, GERP++, PhyloP and SiPhy pathogenicity scores. This database is included in the American College of Medical Genetics and Genomics (ACMG) classification provided by Varsome, and in the filter-based annotation provided by Annovar. The potential clinical impact of stop-gain splicing variants, frameshift, and in-frame insertions and deletions was estimated with Mutation Taster. Each variant was then matched with its classification in publicly-available databases and datasets like the NCBI ClinVar database, the Human Genome Mutation Database (HGMD), NCBI dbSNP, Catalogue of Somatic Mutation In Cancer (COSMIC), The Cancer Genome Atlas (TCGA), VarSome and 1000 Genomes Project. Variants not previously published or documented in online variant databases were considered as novel.

### Detection of TERT promoter mutations by direct sequencing

Mutational status of *TERT* promoter (between − 286 and − 27 from ATG start site) was screened by Sanger sequencing. PCR was carried out with the designed primers [forward: 5'CCCACGTGCGCAGCAGGAC 3'; reverse: 5'CTCCCAGTGGATTCGCGGGC 3'] by using the following cycling conditions: denaturation at 95 °C for 45 s, annealing at 60 °C for 30 s, extension at 72 °C for 30 s. The amplified products underwent sequencing analysis on the Applied Biosystems™ 3500 Series Genetic Analyzer instrument (Thermo Fisher, Foster City, USA) and the sequencing data were investigated by using Geneious Pro 5.6.5 software mapping to the hg19 human reference genome from NCBI gene database.

### Statistical analysis

The relationship between mutations and clinic-pathological features was evaluated using logistic regression analysis with estimation of OR and 95% CI. Semi-quantitative data were analysed by means with Student’s t test or by medians with Mann–Whitney test. *P* values < 0.05 were considered statistically significant. Computations were performed using the R v3.6.2 statistical package. Mutual exclusivity analysis was performed by using the cBioPortal for Cancer Genomics tool^20^ that identify patterns of mutual exclusivity or co-occurrence through a Fisher's exact test^21^. The PCA (Principal Component Analysis) multivariate analysis was performed in order to identify genetic variations among superficial and nodular BCC subgroups. PCA diagram was generated with the *prcomp* R function and plotted with ggplot2^23^.

### Ethics declarations

The study was approved by the local ethical committee (IRB number: 15272/14, Ethical Committee Fondazione Policlinico Universitario A. Gemelli-IRCCS) and the research was performed in accordance with relevant guidelines and regulations stated in the Declaration of Helsinki. The patients in this manuscript have given written informed consent to publication of their case details.

## Supplementary Information

Supplementary Information 1.

Supplementary Information 2.

Supplementary Information 3.

Supplementary Information 4.

## Data Availability

The datasets presented in this study can be found at the online NCBI BioProject repository, https://www.ncbi.nlm.nih.gov/Traces/study/?acc=PRJNA731355, accession PRJNA731355.
