# Supplementary figures and images for: Molecular alterations in basal cell carcinoma subtypes
Source: Sci Rep. 2021 Jun 24;11:13206. doi: 10.1038/s41598-021-92592-3 (PMC8225846; doi:10.1038/s41598-021-92592-3)

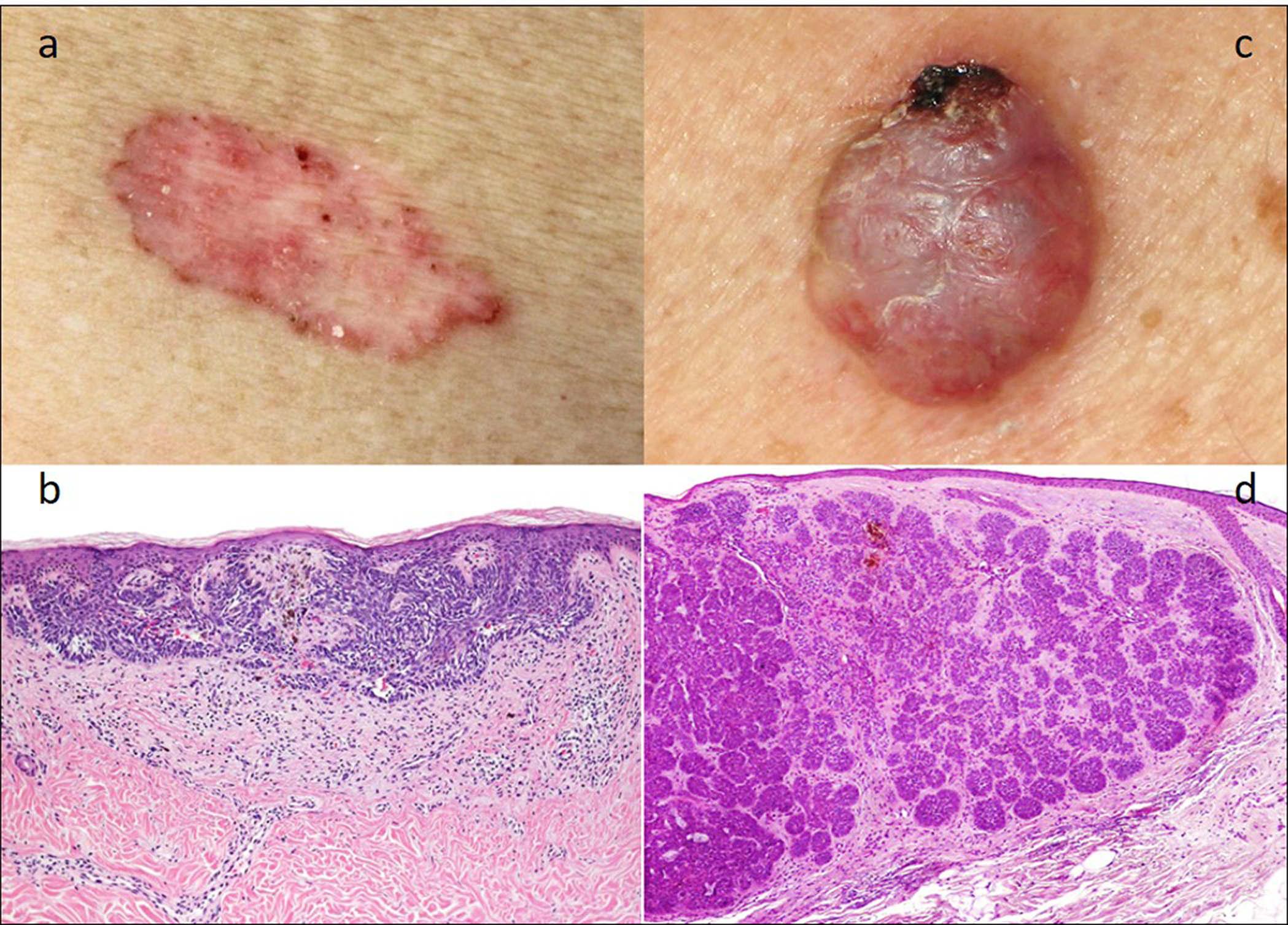

Supplement: Supplementary file 1 — Supplementary Information 1. [file 41598_2021_92592_MOESM1_ESM.jpg]
