# Supplementary material for: Molecular alterations in basal cell carcinoma subtypes
Source: Sci Rep. 2021 Jun 24;11:13206. doi: 10.1038/s41598-021-92592-3 (PMC8225846; doi:10.1038/s41598-021-92592-3)
Supplement: Supplementary file 2 — Supplementary Information 2. [file 41598_2021_92592_MOESM2_ESM.pdf]

Molecular alterations in basal cell carcinoma subtypes

Lucia Di Nardo,[1,2§] Cristina Pellegrini,[3§] Alessandro Di Stefani,[2] Francesco Ricci,[4] Barbara Fossati,[2] Laura Del Regno,[2] Carmine Carbone,[5] Geny Piro,[5] Vincenzo Corbo,[6] Pietro Delfino,[6] Simona De Summa,[7] Maria Giovanna Maturo,[3] Tea Rocco,[3] Giampaolo Tortora,[5,6] Maria Concetta Fargnoli,[3] Ketty Peris[*1,2]

Supplementary material

Supplementary Figure S1. (a-b) Superficial BCC. a) clinical image: flat, pink-to-red, well-circumscribed patches; b) histological image: nests of basaloid cells project from the epidermis. (c-d) Nodular BCC. c) clinical image: smooth, translucent, greyish papule with overlying telangiectasia, pearly border and ulceration; d) histological image: islands of neoplastic basaloid cells with peripheral palisading, extending into the dermis [b,d: Hematoxylin and eosin stain, original magnification x100].

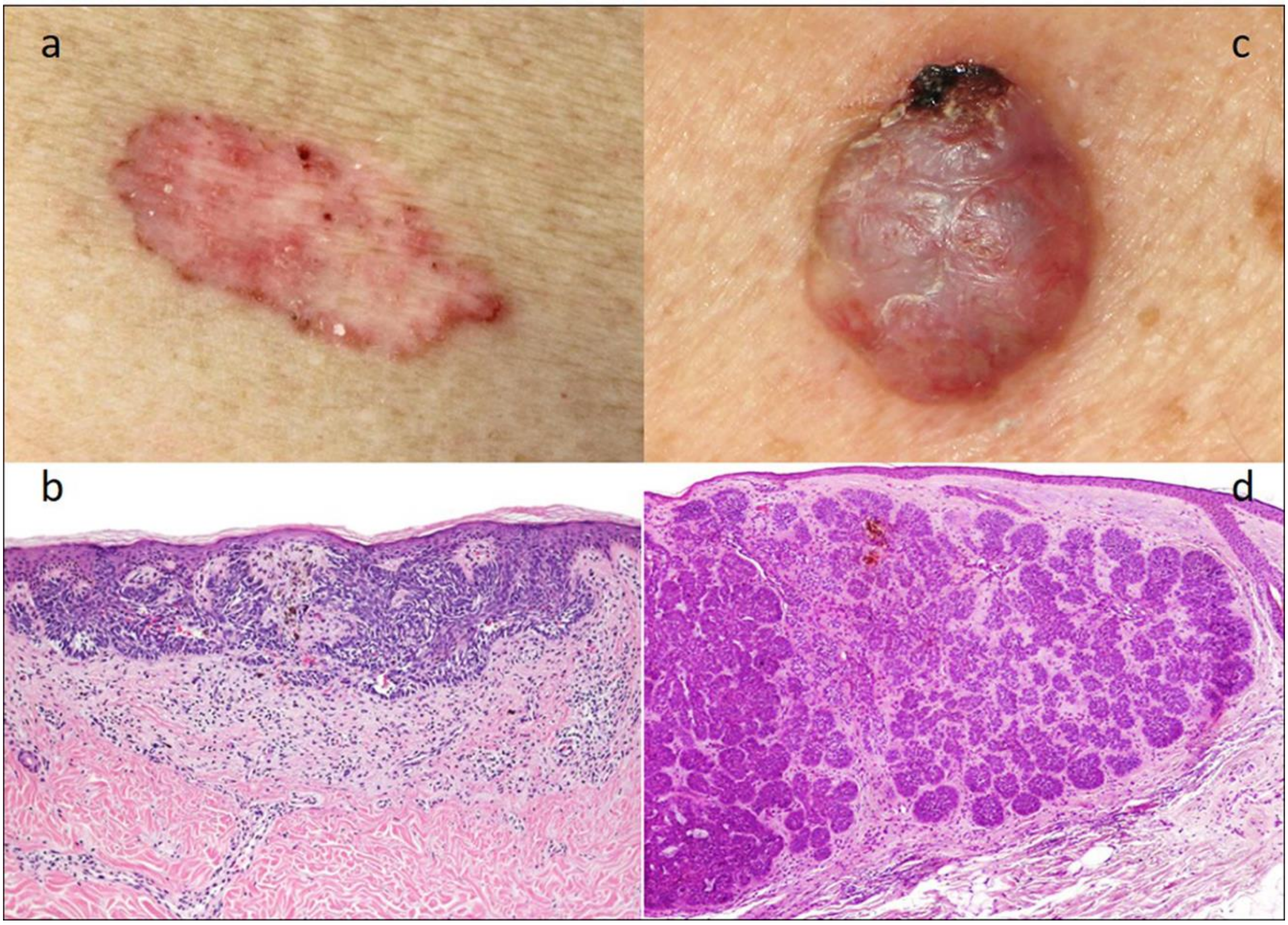

Supplementary Table S1. Human reference genome from NCBI gene database used for sequencing data (CDS: complete coding sequence)

Gene	Genomic ID	Transcript ID	Locus	Chr_Start	Chr_End
**CSMD1**	NC_000008.11	NM_033225	8p23.2	CDS	
**CSMD2**	NC_000001.11	NM_052896	1p35.1-p34.3	CDS	
**DPP10**	NC_000002.12	NM_020868	2q14.1	CDS	
**PTCH1**	NC_000009.12	NM_000264	9q22.32	CDS	
**TP53**	NC_000017.11	NM_000546	17p13.1	CDS	
**NOTCH1**	NC_000009.12	NM_017617	9q34.3	CDS	
**NOTCH2**	NC_000001.11	NM_024408	1p12-p11.2	CDS	
**SMO**	NC_000007.14	NM_005631	7q32.1	CDS	
**DPH3 promoter**	NC_000003.12	NM_206831	3p25.1	16306256	16306755
**ITIH2**	NC_000010.11	NM_002216	10p14	CDS	
**STEAP4**	NC_000007.14	NM_024636	7q21.12	CDS	
**GLI1**	NC_000012.12	NM_005269	12q13.3	CDS	
**TERT promoter**	NC_000005.10	NM_198253	5p15.33	1295071	1295521

Supplementary Table S2. Somatic mutations identified in BCC tumours (file excel)

Supplementary Table S3. *TERT* and *DPH3* promoter mutations identified in the study

**TERT promoter mutation type**	**BCCs**<br>**N= 57 (%)**
c.-146 C>T	19 (33.3)
c.-124 C>T	5 (8.8)
c.-138/-139 CC>TT	3 (5.2)
c.-124/125 CC>TT	1 (1.7)
c.-138 C>T	2 (3.5)
c.-101 C>T	1 (1.7)
c.-102 C>T	1 (1.7)
c.-126 C>T	1 (1.7)
**DPH3 promoter mutations type**	**BCCs**<br>**N= 57 (%)**
c.-121 C>T	19 (33.3)
c.-122 C>T	4 (7.0)
c.-125C>T	2 (3.5)
c.-150C>T	2 (3.5)
c.-121/122 CC>TT	1 (1.7)
c.-189 C>T	1 (1.7)
c.-114 C>T	1 (1.7)
c.-108 C>T	1 (1.7)
c.-76 C>T	1 (1.7)

Mutation names refer to the position from ATG start site (RefSeq NC_000003.12 for *DPH3* promoter and NC_000005.10 for *TERT* promoter).

Supplementary Table S4. Clinical and molecular features of patients and tumours (file excel)
